# Supplementary material for: The squeezed dark nuclear spin state in lead halide perovskites
Source: Nat Commun. 2023 Oct 21;14:6683. doi: 10.1038/s41467-023-42265-8 (PMC10590392; doi:10.1038/s41467-023-42265-8)
Supplement: Supplementary file 1 — Supplementary Information [file 41467_2023_42265_MOESM1_ESM.pdf]

Supplementary Information
"The squeezed dark nuclear spin state in lead halide perovskites"

E. Kirstein, D. S Smirnov, E. A. Zhukov, D. R. Yakovlev, N. E. Kopteva, D. N. Dirin, O. Hordiichuk, M. V. Kovalenko, and M. Bayer

SUPPLEMENTARY NOTE 1: EXPERIMENTAL DETAILS

A. Photoluminescence and time-resolved Kerr ellipticity

A photo of the hybrid organic-inorganic $FAPbBr_3$ perovskite crystal under study is shown in the inset of Supplementary Fig. 1(a). It is a crystal with a size of 5 × 5 × 2 mm^3 and a glossy semi-transparent reddish color. Its low temperature photoluminescence measured at $T = 5$ K has maximum at 2.1776 eV, see Supplementary Fig. 1(a).

In the time-resolved Kerr ellipticity (TRKE) measurements the laser was tuned to the energy of 2.191 eV, resonant with the free exciton. The pump beam was helicity modulated between σ^+/σ^- circular polarization at the frequency of 50 kHz to avoid dynamical nuclear polarization. A typical TRKE dynamics trace measured in $B_V = 0.5$ T magnetic field applied in the Voigt geometry is shown in Supplementary Fig. 1(b). It is contributed by the coherent spin dynamics of charge carriers, which spins are initially oriented by the circularly polarized pump and then precess about the magnetic field. One can clearly see two Larmor precession frequencies, corresponding to electrons (e) and holes (h), which is the typical phenomenology for lead halide perovskite crystals [S1]. We have shown that the coherent spin dynamics in perovskite semiconductors are provided by resident electrons and holes, which are localized in spatially different sites [S2–S4].

The TRKE dynamics were fitted using the function:

$$TRKE = \mathcal{A}_e \cos(\Omega_{L,e} t) \exp(-t/T^*_{2,e}) + \mathcal{A}_h \cos(\Omega_L t) \exp(-t/T^*_{2,h}), \quad (1)$$

as shown by the yellow dashed line in Supplementary Fig. 1(b). $\mathcal{A}_{e(h)}$ are the Kerr ellipticity amplitudes, reflecting the degrees of carrier spin polarization, $\Omega_L = g_h \mu_B B/\hbar$ and $\Omega_{L,e} = g_e \mu_B B/\hbar$ are the Larmor frequencies with the carrier g-factors $g_{e(h)}$, and $T^*_{2,e(h)}$ are the ensemble spin dephasing times. There we show the individual components of the hole (blue line) and the electron (red line) spin dynamics obtained form the fit with the parameters $g_h = +0.4$ and $T^*_{2,h} = 780$ ps for the holes and $g_e = +2.4$ and $T^*_{2,e} = 1080$ ps for the electrons. The assignment of the components is made based on the universal dependence of their g-factors on the band gap energy that we have published recently [S1].

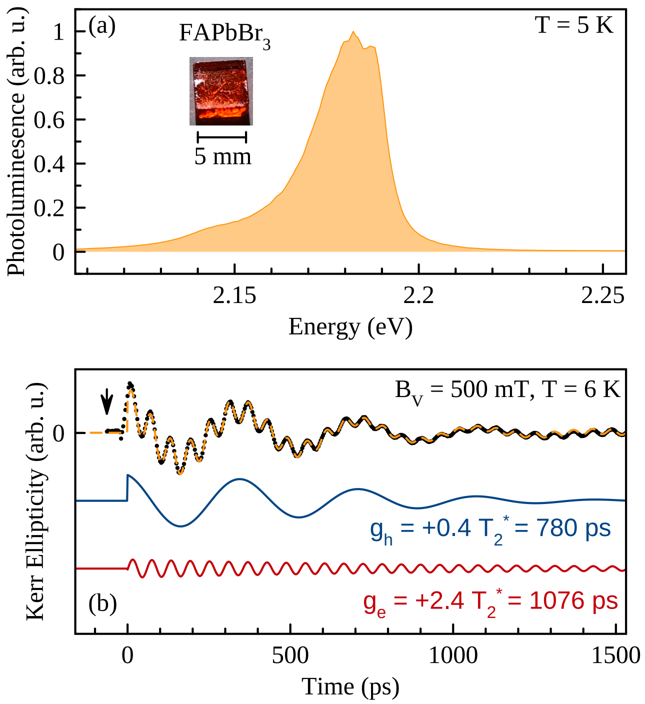

Supplementary Figure 1. (a) Photoluminescence spectrum of the $FAPbBr_3$ crystal measured under continuous-wave excitation with the photon energy of 3.06 eV at $T = 5$ K. Inset shows a photo of the studied crystal. (b) Example of the TRKE dynamics (black line) at the pump-probe energy of 2.191 eV. The pump beam was helicity modulated between σ^+/σ^- circular polarization at the frequency of 50 kHz. Its fit with Supplementary Eq. (1) is shown by the yellow dashed line. The electron and hole spin precession components evaluated from the fit are shown below by the red and blue lines, respectively.

The time delay is fixed at the negative value of −75 ps, marked by the arrow in Supplementary Fig. 1(b), for the measurements of the resonant spin amplification (RSA) and polarization recovery curves (PRCs).

B. Nuclear spin polarization in tilted magnetic field

Using constant pump helicity, additional measurements of the nuclear spin polarization were performed using the same scheme as for Fig. 1. Supplementary Figures 2(a,b) show that the Overhauser field for the electrons does not depend on the strength of the external magnetic field, as expected for dynamic nuclear

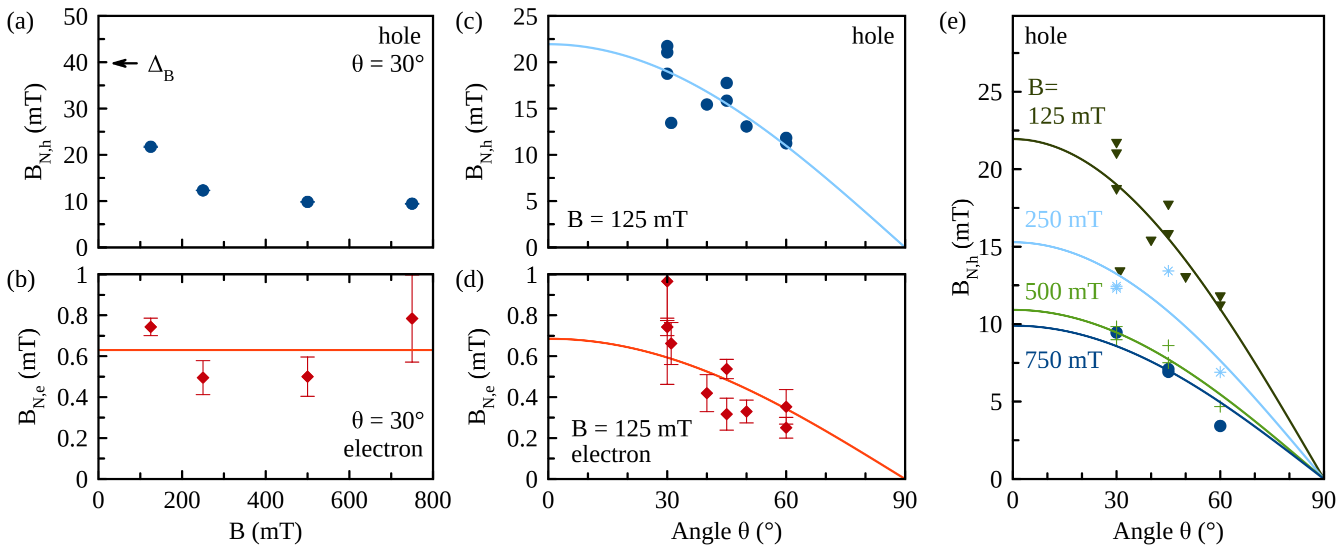

Supplementary Figure 2. (a,b) Overhauser field ($B_{N,e(h)}$) experienced by the hole and electron spins as function of the magnetic field tilted by $\theta = 30°$. Lines are guides to the eye. (c,d) Overhauser fields as function of the tilting angle of the external magnetic field with strength $B = 125\,\mathrm{mT}$. Lines are fits according to $B_N \propto \cos(\theta)$. (e) Same as (c) for different magnetic field strengths. For all panels $T = 1.6\,\mathrm{K}$ and the pump power is 8 mW.

Supplementary Table 1. Isotopes with nonzero nuclear spin in $FAPbBr_3$ with FA = $CH_2(NH)_2$.

Nuclear isotope	abundance [%]	Spin I	NMR γ_N [MHz/T]
^{207}Pb	22.10	1/2	8.882
^{79}Br	50.69	3/2	10.704
^{81}Br	49.31	3/2	11.538
^{1}H	99.98	1/2	42.577
^{13}C	1.07	1/2	10.708
^{14}N	99.63	1	3.078

spin polarization [S5]. The Overhauser field for the holes decreases from 22 mT at B = 150 mT to 12 mT at $B = 250$ mT and then stays about constant at 10 mT up to the magnetic field of 750 mT. Nevertheless, it remains small in comparison with its typical stochastic fluctuation in equilibrium, $\Delta_B = 40$ mT.

In the angular dependence of the Overhauser field for both electron and hole spins, despite a certain scattering of the achieved field value, a dependence according to a cosine-function is found, see Supplementary Figs. 2(c,d). The angular dependence of the Overhauser field experienced by the holes for several magnetic fields is given in Supplementary Fig. 2(e).

C. PRC at constant helicity pumping

In order to analyze the limit of $f_{\mathrm{mod}} \to 0$, we performed additional PRC measurements for constant pump helicity, σ^+ or σ^-. Supplementary Figure 3(a) shows the two corresponding Kerr ellipticity dynamics. They merge with each other when reversing amplitude and magnetic field. In particular, when their amplitudes are summed, the narrow peak around $B_F = 0$ becomes fully compensated and cannot be seen [Supplementary Fig. 3(b)]. We plot also the difference of the signals, which corresponds to the nuclear spin inertia signal at $f_{\mathrm{mod}} = 0$ and compare it with the case of $f_{\mathrm{mod}} = 20$ Hz in Supplementary Fig. 3(c). Generally, the signals are similar, however the latter shows two weak maxima at $B_F \approx \pm 10$ mT around the zero-field dip in agreement with the theoretical modeling, see the green curve in Fig. 3(f).

D. PRC power dependence

Here we present data for the pump power dependence of the PRC signals. In Supplementary Figures 4(a,b) we compare the PRC curves for two different regimes: when the DNSS is formed ($f_{\mathrm{mod}} = 20\,\mathrm{Hz}$) and in absence of the DNSS ($f_{\mathrm{mod}} = 5\,\mathrm{kHz}$).

All curves were evaluated by fits with respect to their amplitude, the ratio of broad dip (A_{b}) to the high field (A_{hf}) amplitude and the dip width, shown in Supplementary Figs. 4(b,c,d), respectively. To start with 5 kHz modulation frequency (full symbols), with increasing pump power the amplitude of the PRC dip and the high field amplitude rise linearly before showing a saturation trend. The narrow dip shows up as a minor peak with magnitude and width near the noise level so that is

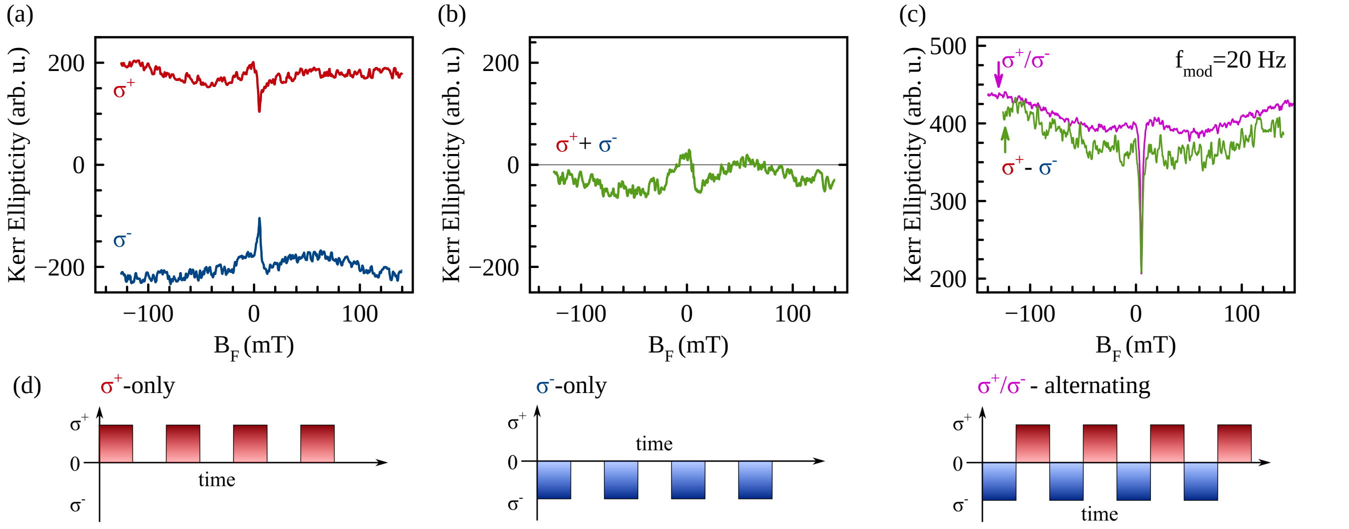

Supplementary Figure 3. (a) PRC with constant pump helicity σ^+ (blue) or σ^- (red). $T = 1.6\,\text{K}$, pump power 6 mW. (b) Sum of the two signals from panel (a). (c) Difference of the two signals from panel (a) (green), corresponding to the nuclear spin inertia signal at $f_{\text{mod}} = 0$, and nuclear spin inertia signal at $f_{\text{mod}} = 20$ Hz (magenta). (d) Scheme of the applied excitation.

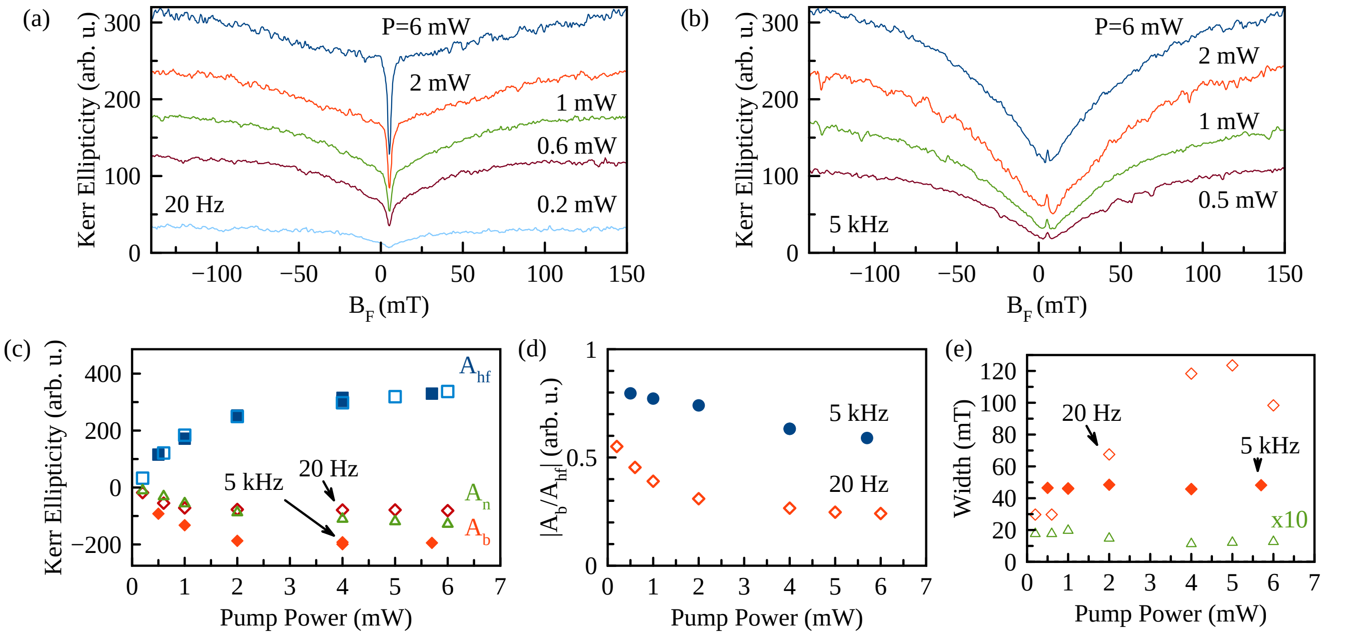

Supplementary Figure 4. PRC at different pump powers P, for (a) $f_{\text{mod}} = 20\,\text{Hz}$ and (b) $f_{\text{mod}} = 5\,\text{kHz}$. The curves are not shifted. (c,d,e) Parameters evaluated from curves (a,b), full symbols for $f_{\text{mod}} = 5\,\text{kHz}$ and open symbols for $f_{\text{mod}} = 20\,\text{Hz}$. (c) Pump power dependence of the amplitudes of the broad A_b and narrow A_n dips (red & dark red diamonds and green triangles, respectively); high field (150 mT) values of the amplitudes A_{hf} (blue & light blue squares). (d) Pump power dependence of the ratio of amplitude of the broad dip and its high field value ($A_{\text{b}}/A_{\text{hf}}$) for $f_{\text{mod}} = 20\,\text{Hz}$ (red) and $f_{\text{mod}} = 5\,\text{kHz}$ (blue). (e) Pump power dependence of the HWHM of the broad (open orange diamonds) and narrow (open green triangles) PRC dips at $f_{\text{mod}} = 20$ Hz, and of the broad dip at $f_{\text{mod}} = 5$ kHz (filled orange diamonds). For all panels $T = 1.6\,\text{K}$.

cannot be evaluated reliably for this frequency. The ratio of the PRC dip to the amplitude at high magnetic fields ($\approx 150\,\text{mT}$) [Supplementary Fig. 4(c)] decreases slightly with increasing pump power. The width stays about constant in Supplementary Fig. 4(d) for 5 kHz modulation.

In contrast, for the low modulation frequency of 20 Hz, the PRC shape changes drastically with varying pump power. Again, the amplitudes at high field (A_{hf}), of the broad dip (A_{b}) and of the narrow dip (A_{n}) rise nonlinearly with a tendency to saturate with increasing pump

power. However, by contrast to the previous case, the ratio A_b/A_{hf} here drops in a nonlinear fashion much stronger. Further, the width of the broad peak broadens significantly with excitation power. The width of the narrow peak remains constant. Note, in both cases of 5 kHz and 20 Hz, the power dependence of the spin signals match each other at the high magnetic field of $B_F = \pm 150$ mT, when the hyperfine interaction plays a minor role, see the blue symbols.

Overall the PRC pump power dependence for the low modulation frequency [Supplementary Fig. 4(a)] is similar to the frequency dependence, shown in Fig. 2(b). As the DNSS formation rate is determined by the flux of angular momentum to the nuclear spin system, this shows that it is also proportional to the flux of angular momentum from exciting photons to a hole in agreement with the theoretical prediction, Supplementary Eq. (20).

E. DNSS at zero magnetic field

Surprisingly, we do not observe the hole spin polarization recovery with decreasing modulation frequency at zero magnetic field, which evidences the fragility of the DNSS in the field range $\lesssim 1$ mT. The exact reason for the DNSS not to form at zero field is unclear so far, but we note that the ODNMR in Fig. 4 has the same linewidth of about 1 mT as the narrow PRC dip. This suggests that the DNSS is destroyed by the nuclear dipole-dipole interactions either between Pb nuclei or with other nuclei such as Br. The weak dependence of the HWHM of this component on the modulation frequency shown in Fig. 2(c) supports this suggestion. Application of the magnetic field with strengths exceeding that of the local fields suppresses this interaction and stabilizes the DNSS.

SUPPLEMENTARY NOTE 2: SIMULATION OF DNSS FORMATION

We describe the nuclear spin dynamics in the pump-probe experiments using the central spin box model. The Hamiltonian of the system reads

$$\mathcal{H} = A\boldsymbol{I}\boldsymbol{S} + \hbar\Omega_L S_z, \tag{2}$$

where $\boldsymbol{S}$ is the hole spin, A is the joint hyperfine interaction constant for all N nuclei in the hole localization volume, $\boldsymbol{I} = \sum_{n=1}^{N} \boldsymbol{I}_n$ is the total nuclear spin composed of the individual spins $\boldsymbol{I}_n$. The hyperfine interaction is assumed here to be isotropic because of the dominant S-type Bloch wave functions at the top of the valence band in perovskites. The same description is valid for electrons in conventional GaAs-like semiconductors. However, in this work on perovskites the hyperfine interaction of the holes plays the main role. We note that the interaction with Br nuclei [S10] can reduce the efficiency of the spin transfer from holes to Pb, which can be absorbed in the renormalization of Γ_0 and ν_0, see below.

A. PRC without DNSS

In equilibrium or at high enough polarization modulation frequencies, the nuclear spin distribution is Gaussian:

$$\mathcal{F}(B_N) = \frac{\exp(-2B_N^2/\Delta_B^2)}{(\sqrt{\pi/2}\Delta_B)^3} \tag{3}$$

with the parameter

$$\Delta_B = \frac{\sqrt{N}A}{g_h\mu_B}, \tag{4}$$

determining the dispersion of the distribution. In these conditions, the PRC is described by [S11]

$$\mathrm{TRKE} = 1 - \frac{2}{3}\frac{\Delta_B^2}{\Delta_B^2 + B^2}. \tag{5}$$

One can see from this expression that the HWHM of the PRC is given by Δ_B, which in combination with Supplementary Eq. (4) yields

$$N = (g_h\mu_B\Delta_B/A)^2. \tag{6}$$

The constant A in the Hamiltonian, Supplementary Eq. (2), is related to the hyperfine coupling constant for a hole localized in a unit cell A_0 by $A = A_0/(N/\beta)$, where $\beta = 0.22$ is the natural abundance of ^{207}Pb. With this we arrive at

$$N = \left(\frac{A_0\beta}{g_h\mu_B\Delta_B}\right)^2, \tag{7}$$

which is used in the main text to determine the number of nuclei N.

B. Nuclear spin dynamics

To describe the nuclear spin dynamics, we note that in Supplementary Eq. (2) the total nuclear spin I is conserved. For N nuclear spins 1/2, the number of possible realizations of spin I is $C_N^{N/2-I} - C_N^{N/2-I-1}$, where C_n^k is the binomial coefficient, and the probability to find the spin I is

$$P_I = \frac{(2I+1)(C_N^{N/2-I} - C_N^{N/2-I-1})}{2^N}. \tag{8}$$

The eigenstates of the system can be labeled by the total angular momentum $F_z = I_z + S_z$, and have the form

$$\Psi_\pm(F_z) = \mathcal{A}_\pm(F_z)\,|F_z + 1/2, \downarrow\rangle + \mathcal{B}_\pm(F_z)\,|F_z - 1/2, \uparrow\rangle\,, \tag{9}$$

where ↑ (↓) corresponds to a hole with spin $S_z = +1/2$ $(-1/2)$ and

$$\mathcal{A}_+(F_z) = -\mathcal{B}_-(F_z) = \frac{\Omega_x}{\sqrt{2\Omega(\Omega+\Omega_z)}}, \tag{10a}$$

$$\mathcal{A}_-(F_z) = \mathcal{B}_+(F_z) = \sqrt{\frac{\Omega+\Omega_z}{2\Omega}}, \tag{10b}$$

with

$$\Omega_x = \frac{A}{\hbar}\sqrt{I(I+1) - F_z^2 + 1/4}, \tag{11a}$$

$$\Omega_y = 0, \tag{11b}$$

$$\Omega_z = \Omega_L + \frac{A}{\hbar}F_z \tag{11c}$$

and

$$\Omega = |\mathbf{\Omega}| = \frac{1}{\hbar}\sqrt{A^2 I(I+1) + \hbar^2\Omega_L^2 + 2AF_z\hbar\Omega_L + A^2/4}. \tag{12}$$

For a given I_z after initialization of the hole in the spin-up state, the wave function is $|I_z, \Uparrow\rangle$, which can be represented as

$$|I_z, \Uparrow\rangle = \alpha\Psi_+(I_z + 1/2) + \beta\Psi_-(I_z + 1/2), \tag{13}$$

where

$$\alpha^2 = \frac{\mathcal{A}_-^2(I_z+1/2)}{\mathcal{A}_+^2(I_z+1/2) + \mathcal{A}_-^2(I_z+1/2)}, \tag{14a}$$

$$\beta^2 = \frac{\mathcal{A}_+^2(I_z+1/2)}{\mathcal{A}_+^2(I_z+1/2) + \mathcal{A}_-^2(I_z+1/2)}. \tag{14b}$$

Then the coherence between the states $\Psi_+(I_z + 1/2)$ and $\Psi_-(I_z + 1/2)$ is lost [S12] on the timescale $T_{2,h}^* \sim \hbar/(AI) \ll T_R$, where T_R is the repetition period of the pump pulses. As a result, the nuclear spin component I_z increases by unity with the probability

$$\begin{aligned} v(I_z+1/2) &= |\alpha\mathcal{A}_+(I_z+1/2)|^2 + |\beta\mathcal{A}_-(I_z+1/2)|^2 \\ &= \frac{2\mathcal{A}_-^2(I_z+1/2)\mathcal{A}_+^2(I_z+1/2)}{\mathcal{A}_-^2(I_z+1/2)+\mathcal{A}_+^2(I_z+1/2)} = \frac{1}{2}\left(\frac{\Omega_x}{\Omega}\right)^2. \end{aligned} \tag{15}$$

Here the argument $I_z + 1/2$ is used to denote the total angular momentum component of the nuclei and the hole. In the limit of classical I, this reduces to $v(I_z + 1/2) = A^2(I_x^2 + I_y^2)/(2|A\boldsymbol{I} + \hbar\mathbf{\Omega}_L|^2)$. For a hole with spin down, the nuclear spin projection reduces by unity with the probability $v(I_z - 1/2)$.

In the limit of many nuclear spins, $N \gg 1$, it is convenient to introduce the distribution function $g(x)$, where $x = I_z/I$, which is normalized such that

$$\int_{-1}^{1} g(x)\mathrm{d}x = 1. \tag{16}$$

It satisfies the Fokker-Planck equation

$$I^2 T_R \frac{\partial g}{\partial t} = P_e(t) I \frac{\partial}{\partial x}(vg) + \frac{\partial}{\partial x}\left(\frac{v}{2}\frac{\partial g}{\partial x}\right), \tag{17}$$

where $v = (1-x^2)/2$. Introducing $\tau = t/(I^2T_R)$ and $\Pi(t) = P_e(t)I$, we obtain

$$\frac{\partial g}{\partial \tau} = \Pi(t)\frac{\partial}{\partial x}(vg) + \frac{\partial}{\partial x}\left(\frac{v}{2}\frac{\partial g}{\partial x}\right). \tag{18}$$

As boundary condition, $g(x)$ should be finite at $x = \pm 1$.

From this description, one can see that the hole spin polarization is a function of the total nuclear spin $\boldsymbol{I}$ [see the definition below Eq. (1) and Supplementary Eq. (2)]. So the correlations between distant nuclear spins also contribute to the observed Kerr rotation signal [S12–S14], which allows us to evidence nuclear spin squeezing and entanglement.

C. Nuclear spin inertia

To describe the nuclear spin inertia, we assume that each pump pulse excites a trion with the probability Γ_0 and after its recombination, the hole spin polarization increases by P_h. According to the optical selection rules, the transverse hole spin components subsequently are erased [S15]. This allows us to neglect the off-diagonal components of the hole and nuclei density matrix. In this case, the nuclear spin state is described by the probabilities P_m for finding the spin I in the state with $I_z = m$. We note that after the hole spin decoherence, the hole spin reduces by a factor of $v(F_z)$, which provides an increase of I_z. Thus, if the polarization modulation period consists of N_{mod} pulses, the signal measured by the nuclear spin inertia method is given by [S16]

$$L = \frac{\left|\sum_{k=1}^{N_{\mathrm{mod}}/2}\sum_{m=-I}^{I} P_m(k)v(m+1/2)\right|}{\left|\sum_{k=1}^{N_{\mathrm{mod}}/2}\exp\left(2\pi \mathrm{i}k/N_{\mathrm{mod}}\right)\right|}, \tag{19}$$

where $P_m(k)$ describes the distribution of $I_z = m$ after k pulses, starting from the beginning of the period, and we assume that it is sufficient to consider only the first half of the period.

This formalism allows us to simulate the evolution of the nuclear spin distribution function under pulsed excitation with modulation of the light helicity and to calculate the nuclear spin inertia signal as function of the

magnetic field and the modulation frequency. Due to the numerical difficulties with the boundary conditions for Supplementary Eq. (18), we have used a finite number of nuclei, namely $N = 60$, as estimated in the main text.

To fit the frequency dependence of the PRC amplitude in Fig. 3(d), we have used $\Gamma_0 = 0.003$ and $P_h = 0.8$. The latter parameter determines the saturation of the PRC amplitude at low modulation frequencies. The DNSS formation rate is given by

$$\nu_0 = \Gamma_0 P_h/(T_R N) = 3.2\,\mathrm{ms}^{-1}, \tag{20}$$

it determines the characteristic time scale of the nuclear spin dynamics, or the rate of Overhauser field fluctuation rotation to the z axis. Thus, all parameters can be reliably determined from the experimental results.

The formation of the DNSS requires the rate ν_0 to be larger than the dephasing rate of the nuclei $1/T_2^n$. It is determined by the local fields and can be estimated from the energy of the nuclear dipole-dipole interactions:

$$\frac{1}{T_2^n} \sim \frac{\hbar(\gamma_N I_n)^2\beta}{a_0^3}, \tag{21}$$

where γ_N is the lead gyromagnetic ratio and $I_n = 1/2$ is the spin of ^{207}Pb nuclei. With the parameters given above ($\gamma_N/(2\pi) = 8.882$ MHz/T, $\beta = 0.221$, $a_0 = 0.6$ nm) we obtain $T_2^n \sim 120$ ms, which is indeed much larger than the DNSS formation time $1/\nu_0$. So the nuclear spin dynamics is driven mainly by the hyperfine interaction, and the time scale of the appearance of the longitudinal nuclear spin polarization and suppression of the transverse nuclear spin fluctuations is much shorter than this estimation of T_2^n from the strength of the dipole-dipole interaction.

D. Nuclear spin squeezing and entanglement

We characterize the shrinking of the nuclear spin distribution function along the transverse directions by the Kitagawa and Ueda spin squeezing parameter ξ_s [S17, S18], which is defined by

$$\xi_s^2 = \frac{4}{N}\left\langle I_x^2\right\rangle. \tag{22}$$

During the simulation of the nuclear spin inertia, it can be extracted as

$$\xi_s^2 = \frac{4}{N}\left\langle \sum_{m=-I}^{I} P_m \frac{I(I+1)-m^2}{2} \right\rangle, \tag{23}$$

where the angular brackets denote the averaging over the total nuclear spin I with probabilities Supplementary Eq. (8), and P_m symbolizes $P_m(k)$ with $k = 1$, for which the spin squeezing reaches its maximum.

The result of the calculation of the nuclear spin squeezing degree is shown in Fig. 3(e) by the solid line for the same parameters as in panel (d). To find ξ_s from the experimental results, we assume that the nuclear spin dynamics obey the evolution according to Supplementary Eq. (2). Then, following the same procedure as described above, we find the DNSS that corresponds to the experimentally observed amplitude of the PRC at the given frequency and the calculated spin squeezing parameter for this state. The results are shown in Fig. 3(e) by the dots.

For low modulation frequencies we obtain $\xi_s = 0.48$, which is limited by nuclear spin diffusion due to an incomplete hole spin polarization. In the limit of a perfect nuclear spin alignment along the z axis, the largest possible spin squeezing is $\xi_s = 0.44$ because of the quantum fluctuations of the transverse components of the total nuclear spin $I \sim \sqrt{N} \sim 8$. This situation can be called the Heisenberg limit, and it is only by 10% smaller than the experimentally reached value.

The large nuclear spin squeezing suggests deep entanglement between nuclear spins. One of the strict evidences of entanglement is the violation of the generalized spin squeezing inequality

$$\langle I_x^2\rangle + \langle I_y^2\rangle + \langle (I_z - \langle I_z\rangle)^2\rangle \geq \frac{N}{2}. \tag{24}$$

We note, that this represents a particular case of Eq. (2) with $M = N$, which means that there is at least any entanglement in the nuclear spins system.

The PRC directly measures the transverse nuclear spin components $\langle I_x^2 + I_y^2\rangle = \langle I(I+1) - I_z^2\rangle$ (from this relation one can see that $\langle I_x^2 + I_y^2\rangle$ is smallest for the DNSS, where $I_z = I$ [S17]). To check the violation of Supplementary Eq. (24), we consider the upper limit for the longitudinal nuclear spin fluctuations $\langle (I_z - \langle I_z\rangle)^2\rangle \leq N/4$, which corresponds to uncorrelated individual nuclear spins. From the definition in Supplementary Eq. (22) we obtain that states with $\xi_s \leq 1/\sqrt{2} = 0.707$ are entangled. This boundary is shown in Fig. 3(e) by the blue dashed line.

For the lowest polarization modulation frequency of $f_{\mathrm{mod}} = 100$ Hz, the PRC amplitude $[L(B_{\max}) - L(B = 0)]/L(B = 0) = 0.054$ corresponds to $I_x^2 + I_y^2 = 0.054 I^2$, which after averaging over I yields $\langle I_x^2 + I_y^2\rangle = 0.04N$. Combined with the upper boundary for the longitudinal spin fluctuations we obtain

$$\langle I_x^2\rangle + \langle I_y^2\rangle + \langle (I_z - \langle I_z\rangle)^2\rangle \leq 0.29N, \tag{25}$$

which violates Supplementary Eq. (24) almost by a factor of two. In particular, using Eq. (2) we find that the achieved DNSS is at least equivalent to an $M = 0.58N = 35$-body entanglement among the $N = 60$ nuclear spins.

This is a very conservative estimate mainly because of the assumption of unsuppressed longitudinal spin fluctuations. According to our model of DNSS formation, at

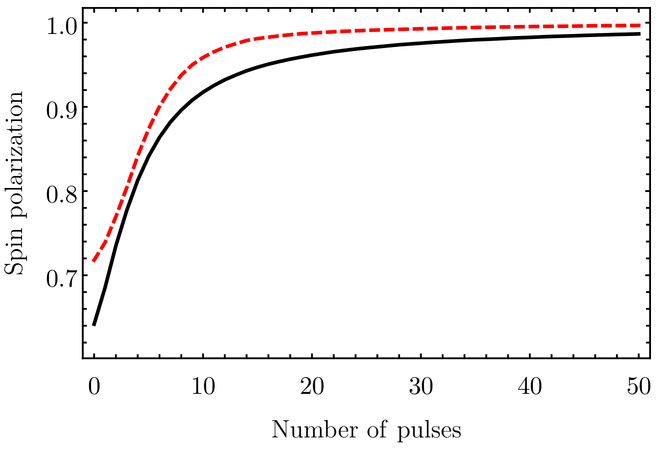

Supplementary Figure 5. Hole spin polarization before the next pump pulse arrival as a function of the number of circularly polarized pulses calculated for the inhomogeneous (black solid curve) and homogeneous (red dashed curve) hyperfine interaction for 10 nuclear spins and $\tau A/\hbar = 17$ (exact value of this parameter is not important as long as it is large).

the lowest polarization modulation frequency the DNSS steady state is reached, where the depth of entanglement is limited by the conservation of I and by nuclear spin diffusion. For this state, the calculation of the nuclear spin fluctuations yields an $M = 19$-body entanglement. We recall that the smaller M is, the deeper is the entanglement.

E. Comparison with the general central spin model

The box model has the advantage of simplicity, however it can underestimate the nuclear spin polarization degree [S19, S20]. In order to compare the general central spin model with the box model, we consider in this subsection $N = 10$ nuclear spins in the vicinity of a hole bound by a zero range potential. The distribution of the hyperfine coupling constants is exponential in this case and we take its average to be equal to the parameter A in the Hamiltonian of the box model. Then we consider hole spin orientation by the train of circularly polarized pulses with the period τ. The hole spin polarization before the next pulse for $A\tau/\hbar \gg 1$ is limited by the hole spin dephasing in the nuclear field. We show it as a function of the number of applied pulses in Supplementary Fig. 5. For many pulses, the spin polarization tends to 1, which is analogous to the disappearance of the dip in the PRC. One can see that this dependence is similar for the homogeneous and inhomogeneous hyperfine interaction.

Let us explain the similarity of the results obtained in the two models. (i) In the case of the box model, the nuclear spin polarization saturates at a small value $\sim 1/\sqrt{N}$, in this case the fluctuations of the total nuclear spin are all rotated towards the z axis. In order to rotate a typical fluctuation of $I \sim \sqrt{N}$ one needs $\sim \sqrt{N}$ pulses (for $A\tau/\hbar \gg 1$). Afterwards, the nuclei are in the DNSS. At the same time, the hole spin, which is oriented along this axis by every optical pulse, does not precess, so there is no dephasing. (ii) For the inhomogeneous hyperfine interaction, a similar number of pulses $\sqrt{N}$ rotates the fluctuations of the Overhauser field towards the z axis, which leads to the formation of the DNSS and suppression of the transverse fluctuations of the Overhauser field. However, afterwards the nuclear spin polarization continues to increase due to the slow destruction of the DNSS [S20]. After a large number of pulses, the spin polarization can be large, of the order of 100% in contrast to the box model. Accordingly, the black curve in Supplementary Fig. 5 increases for $N \sim 3$ as fast as the red one, but then it saturates slower than the red one.

In the nuclear spin inertia measurements, we observe the suppression of the hole spin dephasing and the absence of significant nuclear spin polarization. This corresponds to time scales, when the fluctuations of the Overhauser field are rotated towards the optical axis, but a significant nuclear spin polarization is not reached. So the box model and the general central spin model give similar results on this time scale, which demonstrates applicability of the box model to the description of the nuclear spin squeezing and formation of the DNSS.

SUPPLEMENTARY REFERENCES

[S1] E. Kirstein, D. R. Yakovlev, M. M. Glazov, E. A. Zhukov, D. Kudlacik, I. V. Kalitukha, V. F. Sapega, G. S. Dimitriev, M. A. Semina, M. O. Nestoklon, E. L. Ivchenko, N. E. Kopteva, D. N. Dirin, O. Nazarenko, M. V. Kovalenko, A. Baumann, J. Höcker, V. Dyakonov, and M. Bayer, The Landé factors of electrons and holes in lead halide perovskites: Universal dependence on the band gap, Nat. Commun. **13**, 3062 (2022)

[S2] V. V. Belykh, D. R. Yakovlev, M. M. Glazov, P. S. Grigoryev, M. Hussain, J. Rautert, D. N. Dirin, M. V. Kovalenko, and M. Bayer, Coherent spin dynamics of electrons and holes in $CsPbBr_3$ perovskite crystals, Nat. Commun. 10 , 673 (2019).

[S3] E. Kirstein, D. R. Yakovlev, E. A. Zhukov, J. Höcker, V. Dyakonov, and M. Bayer, Spin dynamics of electrons and holes interacting with nuclei in $MAPbI_3$ perovskite single crystals, ACS Photon. **9**, 1375 (2022).

[S4] E. Kirstein, D. R. Yakovlev, M. M. Glazov, E. Evers, E. A. Zhukov, V. V. Belykh, N. E. Kopteva, D. Kudlacik, O. Nazarenko, D. N. Dirin, M. V. Kovalenko, and M. Bayer, Lead-dominated hyperfine interaction impacting the carrier spin dynamics in halide perovskites, Advanced Materials **34**, 2105263 (2022).

[S5] M. M. Glazov, *Electron and nuclear spin dynamics in semiconductor nanostructures* (Oxford University Press, Oxford, 2018).

[S6] I. A. Yugova, M. M. Glazov, D. R. Yakovlev, A. A. Sokolova, and M. Bayer, Coherent spin dynamics of electrons and holes in semiconductor quantum wells and

quantum dots under periodical optical excitation: Resonant spin amplification versus spin mode locking, Phys. Rev. B **85**, 125304 (2012).
[S7] V. K. Kalevich, Optically induced nuclear magnetic resonance in semiconductors, Sov. Phys. Solid State **28**, 1947 (1986).
[S8] E. A. Zhukov, A. Greilich, D. R. Yakovlev, K. V. Kavokin, I. A. Yugova, O. A. Yugov, D. Suter, G. Karczewski, T. Wojtowicz, J. Kossut, V. V. Petrov, Yu. K. Dolgikh, A. Pawlis, and M. Bayer, All-optical NMR in semiconductors provided by resonant cooling of nuclear spins interacting with electrons in the resonant spin amplification regime, Phys. Rev. B **90**, 085311 (2014).
[S9] R. W. Mocek, V. L. Korenev, M. Bayer, M. Kotur, R. I. Dzhioev, D. O. Tolmachev, G. Cascio, K. V. Kavokin, and D. Suter, High-efficiency optical pumping of nuclear polarization in a GaAs quantum well, Phys. Rev. B **96**, 201303 (2017).
[S10] F. Valadares, I. Guilhon, L. K. Teles, and M. Marques, electronic structure panorama of halide perovskites: Approximated DFT-1/2 quasiparticle and relativistic corrections, J. Phys. Chem. C **124**, 18390 (2020).
[S11] D. S. Smirnov, E. A. Zhukov, D. R. Yakovlev, E. Kirstein, M. Bayer, and A. Greilich, Spin polarization recovery and Hanle effect for charge carriers interacting with nuclear spins in semiconductors, Phys. Rev. B **102**, 235413 (2020).
[S12] I. A. Merkulov, A. L. Efros, and M. Rosen, Electron spin relaxation by nuclei in semiconductor quantum dots, Phys. Rev. B **65**, 205309 (2002).
[S13] A. V. Shumilin and D. S. Smirnov, Nuclear spin dynamics, noise, squeezing, and entanglement in box model, Phys. Rev. Lett. **126**, 216804 (2021).
[S14] A. V. Shumilin and D. S. Smirnov, Nuclear spin dynamics and noise in anisotropic large box model, Phys. Solid. State **64**, 200 (2022).
[S15] I. A. Yugova, M. M. Glazov, E. L. Ivchenko, and A. L. Efros, Pump-probe Faraday rotation and ellipticity in an ensemble of singly charged quantum dots, Phys. Rev. B **80**, 104436 (2009).
[S16] D. S. Smirnov, E. A. Zhukov, E. Kirstein, D. R. Yakovlev, D. Reuter, A. D. Wieck, M. Bayer, A. Greilich, and M. M. Glazov, Theory of spin inertia in singly charged quantum dots, Phys. Rev. B **98**, 125306 (2018).
[S17] J. Ma, X. Wang, C. P. Sun, and F. Nori, Quantum spin squeezing, Phys. Rep. **509**, 89 (2011).
[S18] M. Kitagawa and M. Ueda, Squeezed spin states, Phys. Rev. A **47**, 5138 (1993).
[S19] A. Imamoḡlu, E. Knill, L. Tian, and P. Zoller, Optical pumping of quantum-dot nuclear spins, Phys. Rev. Lett. **91**, 017402 (2003).
[S20] H. Christ, J. I. Cirac, and G. Giedke, Quantum description of nuclear spin cooling in a quantum dot, Phys. Rev. B **75**, 155324 (2007).
